# Association of Number of Physician Postgraduate Years With Patient Intubation Outcomes in the Emergency Department

**DOI:** 10.1001/jamanetworkopen.2022.6622

**Published:** 2022-04-08

**Authors:** Tadahiro Goto, Shojiro Oka, Hiroshi Okamoto, Yusuke Hagiwara, Hiroko Watase, Kohei Hasegawa

**Affiliations:** 1 Department of Clinical Epidemiology and Health Economics, School of Public Health, University of Tokyo, Tokyo, Japan; 2 TXP Medical Co Ltd, Tokyo, Japan; 3 Department of Emergency Medicine, Okinawa Prefectural Chubu Hospital, Okinawa, Japan; 4 Department of Critical Care Medicine, St Luke’s International Hospital, Tokyo, Japan; 5 Department of Emergency Medicine, Tokyo Metropolitan Children’s Medical Center, Tokyo, Japan; 6 Department of Emergency and General Internal Medicine, Fujita Health University, Toyoake, Japan; 7 Department of Emergency Medicine, Massachusetts General Hospital, Boston

## Abstract

This cohort study examines the association between the number of physician postgraduate years and intubation outcomes among patients undergoing airway management in the emergency department.

## Introduction

Evidence suggests that tracheal intubation performed by residents may be a risk factor for poor intubation-related patient outcomes.^1,2^ However, the incidence of intubation-related adverse events is relatively low,^3^ precluding investigators from elucidating the relationship between training levels and intubation performance in the emergency department (ED). This knowledge gap has hindered efforts to develop a consensus on the degree to which between-physician differences in intubation success and adverse event rates should be permitted for the safety of critically ill patients, while providing sufficient training opportunities for residents. We examined the association between the intubator’s number of postgraduate years (PGYs) and intubation outcomes in a large multicenter prospective study of ED patients who underwent airway management.

## Methods

This analysis used data from the second Japanese Emergency Airway Network prospective cohort study, designed to characterize airway management in EDs across Japan. The study setting, methods, and variables measured were described previously,^1^ and further details are provided in the eMethods in the Supplement. The institutional review board at each participating institution approved the study and waived the need for informed consent. This study followed the STROBE reporting guideline.

We included data from 15 institutions for all patients who underwent emergency intubation between 2012 and 2019. Outcome measures were first-pass success and intubation-related adverse events (overall, major, and minor).^1^ To determine the association of physician PGYs with each intubation outcome, we constructed multivariable linear and logistic regression models. All *P* values of <.05 were considered statistically significant (2-tailed).

## Results

Of 11 297 eligible patients, the median age was 71 years (IQR, 56-81) and 7001 patients (62%) were men (Table 1). Overall, 4480 patients (40%) underwent intubation by transitional-year residents (PGY1 to PGY2) and 3588 (31%) underwent intubation by physicians (PGY3 to PGY5). The overall first-pass success rate was 71%. Intubations performed by a less experienced physician had a significantly lower first-pass success rate (Table 2), with an adjusted risk difference of −23% (95% CI, −30% to −16%) for PGY1 residents compared with PGY6 or greater physicians. These associations remained in logistic regression models (adjusted odds ratio, 0.30 [95% CI, 0.22 to 0.41] for PGY1 residents vs PGY6 or greater physicians). Overall, 1802 patients (16%) had intubation-related adverse events. A lower number of PGYs was also associated with a higher rate of any adverse events (adjusted risk difference, 7% [95% CI, 3% to 11%] for PGY1 residents vs PGY6 or greater physicians).

**Table 1.** Characteristics and Airway Management of Patients Receiving Intubation in the Emergency Department^a^

Variable	Values
No. of patients	11 297
Patient characteristics	
Age, median (IQR), y^b^	71 (56-81)
Sex	
Men	7001 (62)
Women	4296 (38)
BMI, median (IQR)	22 (20-25)
≤18.4	6513 (58)
18.5-24.9	1793 (16)
25.0-29.9	1743 (15)
≥30.0	522 (5)
Missing	726 (6)
Primary indications	
Medical	
Cardiac arrest	3951 (35)
Altered mental status	2430 (22)
Airway obstruction or respiratory failure	2092 (19)
Shock	1149 (10)
Other medical condition	76 (1)
Trauma	
Cardiac arrest	467 (4)
Without cardiac arrest	1132 (10)
Components of the modified LEMON score^c^	3670 (32)
Look externally	614 (5)
Interincisor distance	227 (2)
Thyroid-to-hyoid distance	2127 (19)
Obstruction	800 (7)
Neck mobility	1171 (10)
Airway management method^d^	
Rapid sequence intubation	3643 (53)
Sedation without paralytic agents	1179 (17)
No medication	1616 (23)
Other^e^	441 (6)
Devices	
Direct laryngoscope	7321 (65)
Video laryngoscope	3708 (33)
Other	268 (2)
No. of intubator PGYs	
1	1259 (11)
2	3221 (29)
3	1498 (13)
4	1183 (10)
5	907 (8)
≥6	3229 (29)
ED visit year	
2012 (from April 2012)	979 (9)
2013	1735 (15)
2014	1639 (14)
2015	1567 (14)
2016	1790 (16)
2017	1710 (15)
2018	1373 (12)
2019 (until May 2019)	504 (4)

Abbreviations: BMI, body mass index (calculated as weight in kilograms divided by height in meters squared); ED, emergency department; LEMON, look externally, interincisor distance, thyroid-to-hyoid distance, obstruction, and neck mobility; PGY, postgraduate year.

^a^ Data are presented as number (%) unless indicated otherwise.

^b^ Age was unknown for 22 patients.

^c^ Each component of the modified LEMON score had records that were unknown, including 340 (3%) for look externally, 2634 (23%) for interincisor distance, 2599 (23%) for thyroid-to-hyoid distance, 399 (4%) for obstruction, and 529 (5%) for neck mobility.

^d^ Airway management methods used for 6879 patients without cardiac arrest.

^e^ Defined as intubation using topical anesthesia or paralytic agents without sedative agent.

**Table 2.** Adjusted Associations of Postgraduate Year With Intubation-Related Outcomes in the Emergency Department

Outcome, No. of PGYs	No. of patients	Event rate, %	Adjusted risk difference, % (95% CI)^a^	*P* value	Adjusted OR (95% CI)^b^	*P* value
First-pass success						
1	1259	60	−23 (−30 to −16)	<.001	0.30 (0.22 to 0.41)	<.001
2	3221	62	−21 (−23 to −18)	<.001	0.32 (0.28 to 0.37)	<.001
3	1498	72	−12 (−14 to −9)	<.001	0.50 (0.42 to 0.59)	<.001
4	1183	75	−9 (−12 to −5)	<.001	0.58 (0.47 to 0.70)	<.001
5	907	78	−6 (−8 to −3)	<.001	0.70 (0.59 to 0.84)	<.001
≥6	3229	82	[Reference]	NA	[Reference]	NA
Any adverse events						
1	1259	17	7 (3 to 11)	.001	1.78 (1.31 to 2.41)	<.001
2	3221	17	5 (1 to 8)	.004	1.45 (1.11 to 1.90)	.006
3	1498	18	3 (1 to 5)	.005	1.29 (1.06 to 1.57)	.01
4	1183	15	1 (−4 to 6)	.73	1.09 (0.73 to 1.64)	.66
5	907	17	2 (−1 to 6)	.15	1.23 (0.94 to 1.60)	.13
≥6	3229	14	[Reference]	NA	[Reference]	NA
Major adverse events^c^						
1	1259	5	2 (1 to 4)	.01	1.79 (1.10 to 2.93)	.02
2	3221	5	1 (0 to 1)	.29	1.13 (0.92 to 1.39)	.25
3	1498	8	1 (−1 to 2)	.30	1.14 (0.91 to 1.42)	.25
4	1183	7	−1 (−2 to 1)	.37	0.90 (0.70 to 1.16)	.41
5	907	9	2 (0 to 3)	.08	1.26 (0.98 to 1.63)	.07
≥6	3229	6	[Reference]	NA	[Reference]	NA
Minor adverse events^d^						
1	1259	10	4 (1 to 7)	.02	1.71 (1.11 to 2.64)	.01
2	3221	10	4 (2 to 6)	<.001	1.78 (1.25 to 2.53)	.001
3	1498	7	2 (0 to 4)	.06	1.37 (0.96 to 1.95)	.09
4	1183	6	1 (−2 to 4)	.60	1.15 (0.62 to 2.14)	.65
5	907	6	2 (−1 to 4)	.27	1.30 (0.78 to 2.17)	.31
≥6	3229	6	[Reference]	NA	[Reference]	NA

Abbreviations: NA, not applicable; OR, odds ratio; PGY, postgraduate year.

^a^ Linear regression models adjusting for age, sex, body mass index (calculated as weight in kilograms divided by height in meters squared), primary indications, intubation methods, intubation devices, intubation difficulty (each component of the modified LEMON score: look externally, interincisor distance, thyroid-to-hyoid distance, obstruction, and neck mobility), and visit year, accounting for patient clustering within each hospital. Robust SEs were used to accommodate for heteroscedasticity.

^b^ Logistic regression models adjusted for age, sex, body mass index, primary indications, intubation methods, intubation devices, intubation difficulty (each component of the modified LEMON score: look externally, interincisor distance, thyroid-to-hyoid distance, obstruction, and neck mobility), and visit year, accounting for patient clustering within each hospital. Robust SEs were used to accommodate for heteroscedasticity.

^c^ Major adverse events include cardiac arrest, dysrhythmia, hypotension, hypoxemia, unrecognized esophageal intubation, pneumothorax, and regurgitation.

^d^ Minor adverse events include esophageal intubation with early recognition, airway trauma, dental or lip trauma, and mainstem bronchus intubation.

## Discussion

In this study, 8068 patients (71%) were intubated by residents (PGY1 to PGY5). Therefore, our findings present critical issues that should be addressed to improve patient safety in the ED. Our large multicenter cohort study adds to earlier findings^2^ by suggesting an association between intubations performed by residents and poorer intubation outcomes. To our knowledge, few studies have investigated the association between physician training level and adverse events,^4^ potentially because of the low incidence of adverse events.^3^ The association with adverse events in our study is plausible because less experienced physicians may take longer to intubate a patient and may apply extra force to oral structures.^5^ Although there is currently no consensus on the degree of the differences in success rate by PGYs that can be allowed for patient safety, the approximately 20% lower success rate by PGY1 physicians observed here is not acceptable for ED patients. To improve intubation skills and patient outcomes, several approaches have been proposed, including training in the operating room setting, simulation-based curricula, and close supervision by attending physicians.^6^

Our study has several limitations, including the appropriateness of the use of PGYs as a marker for training levels, unmeasured confounders (eg, intubation resources, neck circumstance), and limited generalizability to other ED settings. Regardless, because a large proportion of patients were intubated by residents, our findings underscore the importance of improving both resident training and current airway management strategies for critically ill patients in the ED.

## Notes

Supplement. **eMethods.** Supplementary Methods
eReferences
