# Supplementary material for: Association of Number of Physician Postgraduate Years With Patient Intubation Outcomes in the Emergency Department
Source: JAMA Netw Open. 2022 Apr 8;5(4):e226622. doi: 10.1001/jamanetworkopen.2022.6622 (PMC8994121; doi:10.1001/jamanetworkopen.2022.6622)
Supplement: Supplement. — eMethods. Supplementary Methods eReferences [file jamanetwopen-e226622-s001.pdf]

Supplementary Online Content

Goto T, Oka S, Okamoto H, Hagiwara Y, Watase H, Hasegawa K. Association of number of physician postgraduate years with patient intubation outcomes in the emergency department. *JAMA Netw Open*. 2022;5(4):e226622. doi:10.1001/jamanetworkopen.2022.6622

eMethods. Supplementary Methods
eReferences

This supplementary material has been provided by the authors to give readers additional information about their work.

© 2022 Goto T et al. *JAMA Network Open*.

eMethods. Supplementary Methods

Association of postgraduate years and intubation outcomes in the emergency department

Study design and settings

This is an analysis of the data from a prospective cohort study—the second Japanese Emergency Airway Network (JEAN-2) study that was designed to characterize the current airway management in EDs across Japan. The study setting, methods of data collection, and measured variables were previously described.[1-3] Briefly, JEAN-2 is a consortium of 15 academic and community EDs from different regions across Japan. These EDs have a median of 28,000 visits annually (range, 14,000-65,000), and pediatric patients are treated in all EDs. All institutions have affiliated emergency medicine residency programs and are staffed by emergency medicine attending physicians. Transitional year residents (PGYs 1 and 2) who rotate EDs also participate in airway management. At the discretion of the ED attending physician, intubations are performed by ED attending physicians, emergency medicine resident physicians, or transitional-year residents rotating the ED. In specific cases (e.g., infants aged ≤2 years, patients with congenital anomaly, patients with stroke, difficult intubation), intubations may be performed by other specialties. The individual protocols regarding the policy and procedures for ED airway management are maintained by each institution. This study followed the STrengthening the Reporting of OBservational studies in Epidemiology (STROBE) reporting guideline for prognostic studies. The institutional review board at each participating institution approved the study and waived the need for informed consent.

© 2022 Goto T et al. *JAMA Network Open*.

Study sample

In the current study, we included all patients who underwent emergency intubation from April 2012 through May 2019. The study excluded patients who underwent intubation with the use of cricothyroidotomy or tracheostomy at the initial intubation attempt (i.e., surgical intubation), patients with unknown airway management status, and data without information on the physician's PGY.

Data collection and processing

After each intubation encounter in the ED, the intubator completed a standardized data collection form. The measured variables were age, sex, principal indication for intubation, methods of intubation, all medications used for intubation, intubation devices, intubator's level of training and specialty, number of attempts, pre- and post-intubation vital signs, intubation success or failure, and intubation-related adverse events.[1] We defined an intubation attempt as a single insertion of the laryngoscope (or other devices) past the teeth.[1-5] An attempt was successful if it resulted in a tracheal tube being placed through the vocal cords, with confirmation by quantitative and/or colorimetric end-tidal carbon dioxide monitoring.

Outcome measures

The outcome measures were the success on the first intubation attempt (first-pass success) and intubation-related adverse events. Intubation-related adverse events included cardiac arrest, dysrhythmia, hypotension, hypoxemia, esophageal intubation, regurgitation, airway trauma, dental or lip trauma, and mainstem bronchus intubation.[2] We further categorized these adverse events into major and minor adverse events, according to prior studies.[2] Major

© 2022 Goto T et al. *JAMA Network Open*.

adverse events comprised cardiac arrest, dysrhythmia, hypotension, hypoxemia, unrecognized esophageal intubation, and regurgitation. Minor adverse events comprised the remaining adverse events, including esophageal intubation with early recognition, airway trauma, dental or lip trauma, and mainstem bronchus intubation.

Statistical analyses

To determine the association of physician's PGY (as a surrogate marker for the airway management training level) with each of the intubation outcomes, we constructed linear (gaussian family and identity link function) and logistic regression (binomial family and logit link function) models with a generalized estimating equation to account for patient clustering within the ED. We assumed an independent correlation structure and used robust standard errors to accommodate heteroscedasticity. In the primary analysis, we used PGY as a categorical variable (PGY-1, PGY-2, PGY-3, PGY-4, PGY-5, and PGY-6+ used as the reference), adjusting for eight potential confounders including age, sex, body mass index (≤18.4, 18.5-24.9, 25.0-29.9, and ≥30 kg/m^2), primary indications (medical cardiac arrest, altered mental status, airway obstruction or respiratory failure, shock, other medical conditions, traumatic cardiac arrest, and traumatic non-cardiac arrest), components of modified LEMON (look externally, inter-incisor distance, thyroid-to-hyoid distance, obstruction, and neck mobility), intubation methods (rapid sequence intubation [RSI], sedation without paralytics, no medication, and others), intubation devices used (direct laryngoscope, video laryngoscope, and others), and year of the ED visit. The covariates were selected based on the clinical plausibility and *a priori* knowledge.[1,2,6-10] In our data, there were missing data in age (n=22, 0.2%) and body mass index (n=726, 6.4%). As for intubation difficulty, there were no missing data with the number and proportion of "unknown"

© 2022 Goto T et al. *JAMA Network Open*.

in each component of the modified LEMON being look externally (n=340, 3.1%), inter-incisor distance (n=2634, 23.3%), thyroid-to-hyoid distance (n=2599, 23.0%), obstruction (n=399, 3.5%), and neck mobility (n=529, 4.7%). We used the missing indicator method to account for these missing data and unknown records in each component of the modified LEMON.

Analyses were conducted with STATA version 15.1 (StataCorp, College Station, TX) and R version 3.6.1 (R Development Core Team, Vienna, Austria). All *P* values of <.05 were considered statistically significant (2-tailed).

eReferences

1. Hasegawa K, Hagiwara Y, Chiba T, et al. Emergency airway management in Japan: Interim analysis of a multi-center prospective observational study. *Resuscitation.* 2012;83(4):428-433.
2. Hasegawa K, Shigemitsu K, Hagiwara Y, et al. Association between repeated intubation attempts and adverse events in emergency departments: an analysis of a multicenter prospective observational study. *Ann Emerg Med.* 2012;60(6):749-754 e742.
3. Goto T, Gibo K, Hagiwara Y, et al. Multiple failed intubation attempts are associated with decreased success rates on the first rescue intubation in the emergency department: a retrospective analysis of multicentre observational data. *Scand J Trauma Resusc Emerg Med.* 2015;23:5.
4. Goto Y, Watase H, Brown CA, 3rd, et al. Emergency airway management by resident physicians in Japan: an analysis of multicentre prospective observational study. *Acute Med Surg.* 2014;1(4):214-221.

© 2022 Goto T et al. *JAMA Network Open*.

5. Sagarin MJ, Barton ED, Chng Y-M, Walls RM. Airway Management by US and Canadian Emergency Medicine Residents: A Multicenter Analysis of More Than 6,000 Endotracheal Intubation Attempts. *Annals of Emergency Medicine.* 2005;46(4):328-336.
6. Buis ML, Maissan IM, Hoeks SE, Klimek M, Stolker RJ. Defining the learning curve for endotracheal intubation using direct laryngoscopy: A systematic review. *Resuscitation.* 2016;99:63-71.
7. Hasegawa K, Hagiwara Y, Imamura T, et al. Increased incidence of hypotension in elderly patients who underwent emergency airway management: an analysis of a multi-centre prospective observational study. *Int J Emerg Med.* 2013;6:12.
8. Jaensson M, Gupta A, Nilsson UG. Gender differences in risk factors for airway symptoms following tracheal intubation. *Acta Anaesthesiol Scand.* 2012;56(10):1306-1313.
9. VanderWeele TJ. Principles of confounder selection. *Eur J Epidemiol.* 2019;34(3):211-219.
10. Hagiwara Y, Watase H, Okamoto H, Goto T, Hasegawa K, Japanese Emergency Medicine Network I. Prospective validation of the modified LEMON criteria to predict difficult intubation in the ED. *Am J Emerg Med.* 2015;33(10):1492-1496.

© 2022 Goto T et al. *JAMA Network Open*.
